# Administration of Selenium Decreases Lipid Peroxidation and Increases Vascular Endothelial Growth Factor in Streptozotocin Induced Diabetes Mellitus

**DOI:** 10.22074/cellj.2017.4161

**Published:** 2017-08-19

**Authors:** Pervin Vural, Gulcan Kabaca, Refia Deniz Firat, Sevgin Degirmecioglu

**Affiliations:** 1 Department of Biochemistry, Istanbul Faculty of Medicine, Istanbul University, Istanbul, Turkey; 2 Department of Oral Surgery, Istanbul Faculty of Dentistry, Istanbul University, Istanbul, Turkey

**Keywords:** Oxidative Stress, Vascular Endothelial Growth Factor, Endothelin 1, Experimental Diabetes Mellitus

## Abstract

**Objective:**

The imbalance in oxidant/antioxidant status plays a pivotal role in diabetes mellitus (DM). Selenium is a integral component of the antioxidant enzyme glutathione peroxidase. Se treatment induces angiogenesis and improves endothelial function through increased expression of vascular endothelial growth factor (VEGF). The aim of this study is to investigate the effect of selenium on oxidative stress, VEGF, and endothelin 1 (ET1) in a DM rat model.

**Materials and Methods:**

We performed an experimental animal study with 64 adult male Wistar-Albino rats. Rats were divided into the following groups (n=8): control (C)7, C21, C+sodium selenite (Se)7, and C+Se21 (control rats), and DM7, DM21, DM+Se7, and DM+Se21 (diabetic rats). Diabetes was induced by 2-deoxy-2-(3-methyl-3-nitrosoureido)- D-glucopyranose [streptozotocin (STZ)]. Three weeks after STZ, DM+Se7 rats received intraperitoneal (i.p.) injections of 0.4 mg/kg Se for 7 days. The DM+Se21 rats received these injections for 21 days. The same dose/duration of Se was administered to the C+Se7 and C+Se21 groups. The remaining rats (C7, C21, DM7, DM21) received physi- ologic saline injections for 7 or 21 days. Ferric reducing antioxidant power (FRAP), malon- dialdehyde (MDA), advanced oxidation protein products (AOPP), and endothelial function markers (VEGF and ET1) in plasma samples were measured.

**Results:**

Diabetic rats (DM7 and DM21) had significantly increased plasma FRAP (P=0.002, P=0.001), AOPP (P=0.024, P=0.01), MDA (P=0.004, P=0.001), and ET1 (P=0.028, P=0.003) levels compared with C7 and C21 control rats. VEGF (P=0.02, P=0.01) significantly decreased in DM7 and DM21 diabetic rats compared with their controls (C7, C21). Se administration reversed the increased MDA and decreased VEGF levels, and lowered plasma glucose levels in the DM+Se7 and DM+Se21 diabetic groups compared with diabetic rats (DM7, DM21). We observed positive correlations between FRAP-AOPP (r=0.460), FRAP-ET1 (r=0.510), AOPP-MDA (r=0.270), and AOPP-ET1 (r=0.407), and a negative correlation between MDA-VEGF (r=-0.314).

**Conclusion:**

We observed accentuated oxidative stress and impaired endothelial function in diabetes. Se treatment reduced lipid peroxidation and hyperglycemia. Se probably improved endothelial dysfunction in diabetic rats because of the increased VEGF levels.

## Introduction

Diabetes mellitus (DM) is a metabolic disorder characterized by hyperglycemia due to defects in insulin secretion or action. Various hyperglycemia- induced biochemical mechanisms are involved in the etiopathogenesis and progression of DM. Hyperglycemia leads to activation of protein kinase C (PKC) and nuclear factor kappa B; activates the polyol pathway; increases formation of advanced glycation end-products (AGEs); and increases flux through the aldose reductase pathway. The commonn mechanism among these pathways is increased oxidative stress which leads to changes in blood flow, vascular permeability, and impaired angiogenesis (1). Oxidative stress arises from an imbalance between radical-generating and radical- scavenging systems. It cause modifications and damage to various molecules, including lipids, proteins, and nucleic acids. Malondialdehyde (MDA) occurs as a result of lipid peroxidation. The advanced oxidation protein product (AOPP) concentration reflects the degree of protein damages caused by oxidative stress. Ferric reducing antioxidant power (FRAP) has been proposed to explore the antioxidant capacity of plasma. 

There is considerable evidence which suggests that endothelial function is impaired in DM and precedes vascular complications (2,3). Vascular endothelial growth factor (VEGF) and endothelin 1 (ET1) are important bioactive substances synthesized in endothelial cells and implicated in diabetic vascular complications (4,5). VEGF is a potent angiogenic factor and a mitogen for vascular endothelium. It is well known that this growth factor initiates the migration and proliferation of endothelial cells and suppress their apoptosis, increases vascular permeability, and initiates monocyte/macrophage chemotaxis (6). Knowledge about VEGF in diabetes and its complications is somewhat controversial. Studies have reported increased (7,8) and decreased (9,10) VEGF levels. VEGF was used successfully for treatment of diabetic limb ischemia (11). On the other hand, inhibition of VEGF has been used in diabetic retinopathy (12). Endothelins (ETs) are vasoactive peptides implicated in the inflammatory process that have important cardiovascular, mitogenic, and neuroregulatory functions (13). There are three isopeptides identified- ET1, 2, and 3. Among the three isopeptides, ET1 is considered the most important vasoconstrictor with angiogenic and mitogenic properties. Besides of its effects on vasal tone, ET1 increases monocyte adhesion, activates macrophages, and promotes vascular smooth muscle cell proliferation and migration (14). Increased plasma ET1 concentrations have been found in diabetic patients (15,16) and in experimental models of diabetes (17,18). 

Selenium is an essential trace element that plays an important role in many physiological mechanisms. It is an integral component of the antioxidant enzyme glutathione peroxidase and selenoproteins with antioxidant properties (19). Deficiency in selenium causes an important reduction in the glutathione peroxidase activity that results in oxidative stress. It has been suggested that changes in selenium homeostasis are related to DM (20). The protective role of selenium administration against oxidative stress and diabetes-induced injury has been reported (21,22). Selenium presumably affects carbohydrate metabolism and has insulin-like actions (20). Additionally, selenium exerts regulatory functions on cellular growrh, survival, cytotoxicity, and transformation. It has been recently shown that selenium treatment induces angiogenesis and improves endothelial dysfunction through increased expression of VEGF in DM (23) and myocardial infarction (24). In contrast, selenium treatment delays the development of various tumors via inhibition of VEGF expression (25). There is only one study in the literature where selenium treatment has been shown to dimish increased ET1 levels in diabetic rats (18). The controversial data about VEGF levels in DM and insufficient reports about the effects of selenium on VEGF and ET1 have encouraged us to investigate the impact of selenium treatment on oxidative stress, VEGF, and ET1 in diabetic rats. 

## Materials and Methods

We performed an experimental animal study with 64 adult male Wistar-Albino rats (weights: 250 ± 15 g) obtained from Istanbul University, Institute for Experimental Medical Research, Turkey. Animals were housed in conventional metallic cages, 4 rats per cage, in a room with the temperature regulated at 21 ± 1˚C and light/dark cycles (12 hours). All animals were given ad libitum access to food and water by a drinking bottle throughout the course of the experiment. Animals received a standard laboratory diet. The Institutional Animal Care and Use Committee of Istanbul University (Project No. 2006/17) approved the experiments. All chemicals were supplied from Sigma (Sigma Chemical Co., St. Louis, MO, USA). 

### Induction of diabetes

A total of 4 groups of randomly selected rats (n=32) received intraperitoneal (i.p.) injections of 65 mg/kg of 2-deoxy-2-(3-methyl-3- nitrosoureido)-D-glucopyranose [streptozotocin (STZ)]. Diabetes was confirmed 48 hours after the STZ injection by the development of hyperglycemia and glycosuria. Hyperglycemia was defined by blood glucose levels more or equal to 250 mg/dL in blood samples obtained from the tail vein of rats and measured with a Glucometer (Accu-Chek Go, Roche, Basel, Sweeden). Urine glucose measurements were performed by urine test strips (Urine Reagent Strip-10 URS-10, Teco Diagnostics, Anaheim, CA, USA). All STZ injected rats became diabetic. Control animals (n=32) received normal saline injections. 

### Treatment protocol

At 3 weeks after STZ administration, we divided the diabetic animals into the following groups (n=8 per group): DM7, DM21, DM+Se7, and DM+Se21. Rats from the DM7 and DM21 groups received physiologic saline solution injections for 7 (DM7) and 21 (DM21) days. The DM+Se7 and DM+Se21 groups were injected with 0.4 mg/kg/i.p. sodium selenite (Se) for 7 and 21 days. We divided the control animals into the following groups (n=8 per group): control (C)7, C21, C+Se7, and C+Se21. The C7 and C21 rars received injections of a physiologic saline solution for 7 (C7) and 21 (C21) days. The C+Se7 and C+Se21 rats received injections of Se (0.4 mg/kg) for 7 and 21 days. At the end of the experimental period (7 and 21 days), all rats were weighed on an EK-i/EW-i scale (A&D Co., Japan). 

### Plasma measurements

On the 7^th^ or 21^st^ days of the experiments and after a 12-hour fast, we aesthetized the rats with injections of ketamine [100 mg/kg/ intramuscular (i.m.)]. Intracardiac blood samples were obtained from all rats and placed in EDTA tubes, after which the rats were sacrificied. Blood samples were centrifuged immediately at 1500 xg (10 minutes, 4˚C) to remove the plasma. Plasma glucose measurements were performed on a Cobas Integra 800 autoanalyzer (Roche Diagnostics, Mannheim, Germany). For the spectrophotometrical measurements, we used an Ultraspec 3000 (Pharmacia Biotech, Biochrom Ltd., Cambridge, UK) and for ELISA, an ELx800 (BioTek Instruments, Inc., Winooski, Vermont, USA).

### Plasma ferric reducing antioxidant power values

We evaluated the plasma antioxidant status using a FRAP assay (26). This assay uses antioxidants as reductants in a redox-linked colorimetric method. In this assay, at a low pH, a ferric-tripyridylazine (Fe3+-TPTZ) complex is reduced to the ferrous form, which can be monitored by measuring the change in absorption at 593 nm. The absorbance change is translated into a FRAP value by relating the change of absornance at 593 nm of the test sample to that of a standard solution with a known FRAP value. The intra-assay coefficient of variation was 4.2% and inter-assay coefficient of variation was 5.2%. 

### Plasma advanced oxidation protein product levels

Measurement of AOPP (oxidation products with characteristic absorbances) are detected spectrophotometrically and calibrated with chloramine-T (27). The absorbance at 340 nm was read after which the concentation of AOPP was expressed in chloramine-T units (µmol/L). The intra-assay coefficient of variation was 4.0% and the inter-assay coefficient of variation was 5.7%. 

### Plasma malondialdehyde levels

We evaluated lipid peroxidation in the plasma by the spectrophotometric method based on the reaction between MDA and thiobarbituric acid (28). The absorbance was read at 535 nm against the blank. The MDA concentrations of the samples were calculated using an extinction coefficient of 1.56×10^5^ M^-1^cm^-1^. MDA levels were expressed in nmol MDA/mL plasma (nmol/mL). The intra- assay coefficient of variation was 4.6% and inter- assay coefficient of variation was 5.5%. 

### Plasma endothelin 1 values 

We estimated plasma ET1 levels with the Phoenix Pharmaceuticals EIA kit (Burlingame, CA, USA). The minimum detectable concentration was 100 pg/mL. The intra-assay coefficient of variation was 5-10% and the inter-assay coefficient of variation was <15%. 

### Plasma vascular endothelial growth factor values

For plasma VEGF estimation, we used the Biosource ELISA kit (Camarillo, CA, USA). The minimum detectable concentration was determined to be 3.7 pg/mL. The intra- and inter- assay coeficients of variation were <10%. 

### Statistical analysis

All statistical analyses were perfomed with International Business Machines (IBM) SPSS statistics for Windows (version 21, SPSS Inc., Chicago, IL, USA). For statistical evaluation, the Kruskall-Wallis, Mann-Whitney-U, student’s t and Spearman correlation tests were used. A P<0.05 was considered to be statistically significant. 

## Results

**Fig.1:**
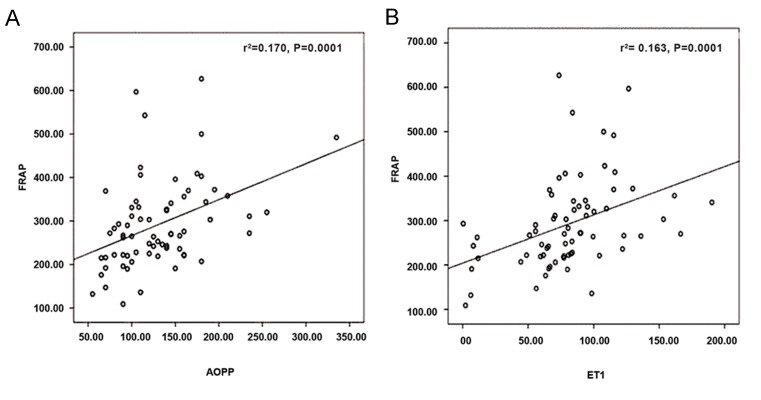
Correlations between FRAP-AOPP and FRAP-ET1 levels. A. FRAP (micromol/L)/AOPP (micromol/L) correction and B. FRAP (micromol/L)/ET1 (pg/ml) correction. FRAP; Ferric reducing antioxidant power, AOPP; Advanced oxidation protein products, ET1; Endothelin 1, and r^2^; Correlation coefficient squared.

**Fig.2:**
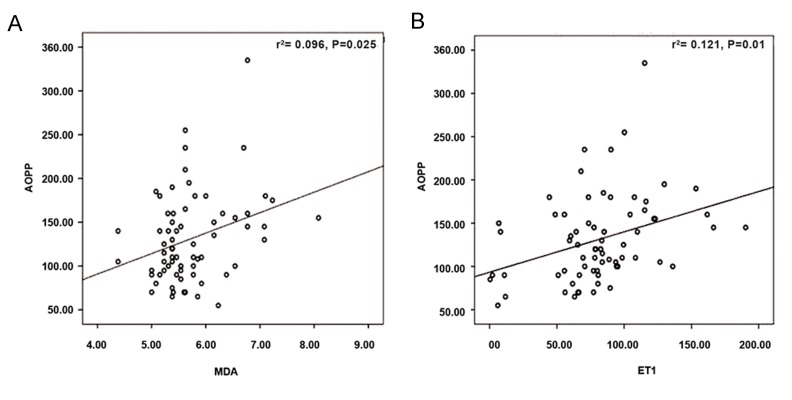
Correlations between AOPP-MDA and AOPP-ET1 levels. A. AOPP (micromol/L)/MDA (nmol/mL) correction and B. AOPP (micromol/L)/ET1 (pg/mL) correction. AOPP; Advanced oxidation protein products, MDA; Malondialdehyde, ET1; Endothelin 1, and r^2^; Correlation coefficient squared.

**Fig.3:**
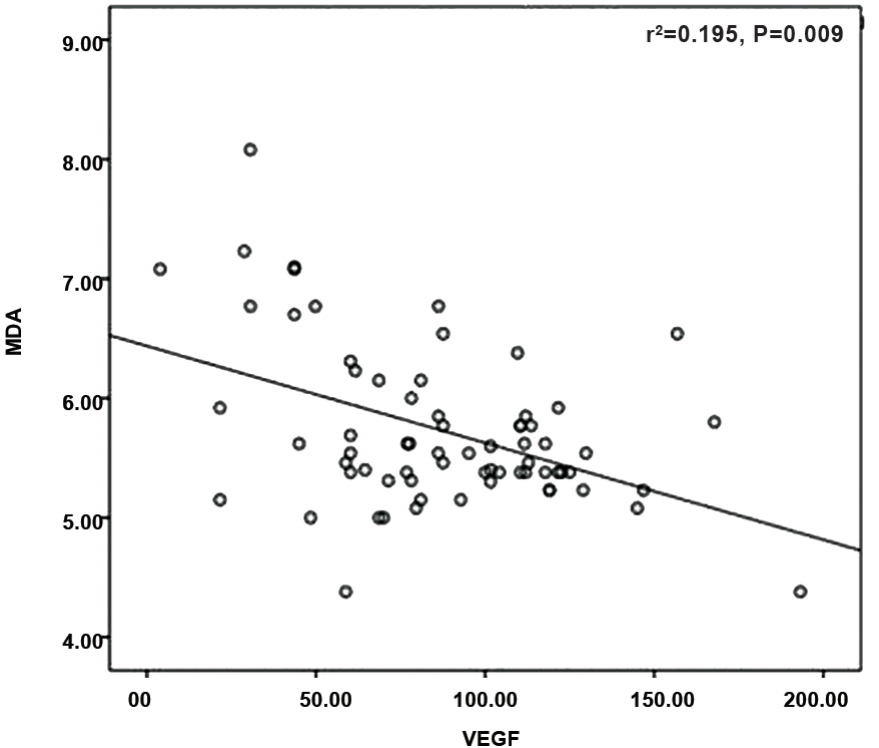
Correlation between MDA (nmol/mL)/VEGF (pg/mL). MDA; Malondialdehyde, VEGF; Vascular endothelial growth factor, and r^2^; Correlation coefficient squared.

Table 1 shows the body weights and plasma glucose levels from the control and diabetic groups. Se treatment lowered plasma glucose levels in the diabetic groups (DM+Se7 and DM+Se21) compared to diabetic rats (DM7 and DM21, P=0.03). Plasma FRAP, AOPP, MDA, VEGF, and ET1 levels in the control and diabetic groups are presented in Table 2. DM7 rats had significantly increased plasma FRAP (P=0.002), AOPP (P=0.024), MDA (P=0.004), and ET1 (P=0.028) levels compared to the C7 control group. DM21 rats also had significantly increased plasma FRAP (P=0.001), AOPP (P=0.01), MDA (P=0.001), and ET1 (P=0.003) levels compared to the C21 control group. VEGF concentrations significantly decreased in DM7 (P=0.02) and DM21 (P=0.01) rats compared to their control groups (C7 and C21). Se treatment did not change FRAP, AOPP, and ET1 in DM, but led to a significant decrease of MDA in the DM+Se7 (P=0.015) and DM+Se21 (P=0.001) and increased VEGF levels in the DM+Se7 (P=0.022) and DM+Se21 (P=0.001) groups compared with diabetic rats (DM7 and DM21). Se administration increased FRAP values in the control C+Se7 (P=0.034) and C+Se21 (P=0.001) rats. Positive correlations existed between FRAP-AOPP (r=0.460, P=0.0001) and FRAP-ET1 [correlation coefficient (r=0.510), P=0.0001, Fig.1] as well as between AOPP-MDA (r=0.270, P=0.025) and AOPP-ET1 (r=0.407, P=0.001, Fig .2). MDA had a negative correlation with VEGF (r=-0.314, P=0.009, Fig .3).

**Table 1:** Mean ± SD body weight and blood glucose levels in control and diabetic rats (n=8 per group)


	Body weight (g)	P value	Blood glucose (mg/dL)	P value

C7	240 ± 10	-	110 ± 15	-
C21	235 ± 15	-	115 ± 18	-
C+Se7	239 ± 9^c^	NS	105 ± 10^c^	NS
C+Se21	241 ± 11^d^	NS	109 ± 15^d^	NS
DM7	200 ± 11^a^	0.01	325 ± 25^a^	0.01
DM21	189 ± 13^b^	0.03	355 ± 20^b^	0.01
DM+Se7	208 ± 10^e^	NS	215 ± 20^e^	0.03
DM+Se21	202 ± 12^f^	NS	220 ± 15^f^	0.03


NS; Not significant, Student’s t test, DM; Diabetes mellitus, C; Control, Se; Sodium selenite, ^a^; DM7 compared to C7, ^b^; DM21 compared to C21, ^c^; C+Se7 compared to C7, ^d^; C+Se21 compared to C21, ^e^; DM+Se7 compared to DM7, and ^f^; DM+Se21 compared to DM21.

**Table 2:** Mean ± SD levels of plasma ferric reducing antioxidant power (FRAP), advanced oxidation protein products (AOPP), malondialdehyde (MDA), vascular endothelial growth factor (VEGF) and endothelin-1 (ET1) in control and diabetic subgroups (n=8 per group)


	FRAP(μmol/L)	P value	AOPP(μmol/L)	P value	MDA(nmol/mL)	P value	VEGF(pg/mL)	P value	ET1(pg/mL)	P value

C7	195.0 ± 36.7	-	116.9 ± 40.0	-	5.5 ± 0.2	-	92.9 ± 33.7	-	718.9 ± 208.6	-
C21	225.3 ± 21.2	-	104.4 ± 32.7	-	5.3± 0.2	-	91.3 ± 24.3	-	684.3 ± 132.2	-
C+Se7	247.8 ± 50.8^c^	0.034	119.4 ± 43.8^c^	NS	5.8 ± 0.5^c^	NS	100.5 ± 23.9^c^	NS	701.1 ± 116.1^c^	NS
C+Se21	341.9 ± 52.4^d^	0.001	109.4 ± 26.4^d^	NS	5.4 ± 0.2^d^	NS	97.1 ± 27.4^d^	NS	831.0 ± 176.7^d^	NS
DM7	318.7 ± 88.9^a^	0.002	209.2 ± 79.9^a^	0.024	6.9 ± 0.8^a^	0.004	36.1 ± 17.0^a ^	0.02	1098.1 ± 338.9^a^	0.028
DM21	368.5 ± 71.6^b^	0.001	151.9 ± 31.3^b^	0.01	6.5 ± 0.6^b^	0.001	67.9 ± 29.9^b^	0.01	1233.0 ± 387.5^b^	0.003
DM+Se7	325.1 ± 54.3^e^	NS	145.4 ± 36.2^e^	NS	5.6 ± 0.7^e^	0.015	92.3 ± 46.8^e ^	0.022	1051.6 ± 297.1^e^	NS
DM+Se21	421.9 ± 158.0^f^	NS	155.0 ± 46.8^f^	NS	5.2 ± 0.4^f^	0.001	128.0 ± 36.8^f ^	0.001	954.3 ± 180.5^f^	NS


NS; Not significant, Kruskall-Wallis and Mann-Whitney U tests, DM; Diabetes mellitus, C; Control, Se; Sodium selenite, ^a^; DM7 were compared to C7, ^b^; DM21 were compared to C21, ^c^; C+Se7 were compared to C7, ^d^; C+Se21 were compared to C2, ^e^; When DM+Se7 were compared to DM7, and ^f^; DM+Se21 were compared to DM21.

## Discussion

The results of this study showed that i. Plasma FRAP, AOPP, MDA, and ET1 levels significantly increased, whereras VEGF significantly decreased in diabetic rats, ii. Se treatment decreased MDA, increased VEGF, and lowered plasma glucose levels; and iii. Positive correlations existed between FRAP-AOPP and FRAP-ET1, as well as between AOPP-MDA and AOPP-ET1. A negative correlation existed between MDA-VEGF. 

It is well known that oxidative stress has an etiological involvement in the development of DM and its complications. Accentuated oxidative stress results in peroxidation of the cell membrane and plasma lipids which are considered primary targets of oxidative stress. The results of numerous clinical (28,34) and experimental (35,37) studies suggest that the lipid peroxidation process is activated in DM. The increased plasma MDA levels in the present study have agreed with others that reported increased lipid peroxidation reflected as MDA. The increased MDA levels could be attributed to the degradation of end- products of polyunsaturated fatty acids of the cell membrane and plasma lipids. In the present study Se administration reversed the increased MDA to the levels found in control rats. This finding supported the outcomes of previous investigations where administration of antioxidants decreased augmented lipid peroxides in DM (35,38,39). 

Together with lipid peroxidation, protein oxidation develops in conditions with accentuated oxidative stress and hyperglycemia. AOPP is a marker of oxidative stress that reflects protein damage (27). According to the results, we have detected elevated AOPP levels in diabetic rats compared with the controls. Increased AOPP levels in types 1 and 2 diabetes (28) that correlate with insulin resistance (33) have been reported. AOPP accumulates over time and correlates with disease duration in type 1 diabetes (31). In addition to changes in lipid structure, oxidative stress results in increased AOPP, probably due to protein damage. A strong correlation between MDA-AOPP, which we have observed in the current study, suggests that oxidative stress affects both plasma lipids and proteins. Se treatment did not change the AOPP levels. 

It is well recognized that a delicate balance exists between oxidative stress and antioxidant defense, and that antioxidant capacity is not completely efficient in living systems. There may be an inverse correlation between oxidant-antioxidant parameters, although the data are controversial (31,35,36). Antioxidant enzymes and vitamins decrease in DM (35,36). In contrast, elevated (29,34) or unchanged (30,37,38) FRAP levels in type 1 diabetes have been reported. We have observed increased FRAP levels in the diabetic rats. FRAP elevation is probably an adaptive compensatory mechanism that counteracts accumulated lipid peroxides and AOPP. The significant correlation between FRAP and AOPP has supported this observation. In our study, Se administration to the diabetic rats did not affect FRAP values. 

However, Se treatment increased FRAP values in the control groups. This increase probably was due to increased antioxidant selenoproteins (39). 

In the present sudy, the diabetic state led to decreased VEGF concentrations that normalized after Se administration. Previous studies showed that plasma VEGF levels increased (7,8) or decreased (9,10) in DM. An association between decreased VEGF and metabolic syndrome was reported (4). VEGF is an angiogenic factor that plays a central role in vasculogenesis and neoangiogenesis, promoting the survival, migration, and proliferation of endothelial cells. VEGF is known to be important for pancreatic β-cell development and function (6). Furthermore, it has been shown that insulin increases VEGF protein expression and secretion via phosphatidylinositol 4,5-biphosphate 3-kinase (PI3-K) and mitogen-activated protein kinase (MAPK) (40). Additionally, in concert with increased VEGF protein expression, insulin stimulates nitric oxide production by the endothelium, and reduced bioavailability of nitric oxide together with oxidative stress results in endothelial dysfunction (3). Hence STZ is a compound that selectively destroys pancreatic β-cells. Therefore, the decrease of VEGF in STZ induced diabetic rats is not a surprise. VEGF plays an important, dual role in diabetic complications (41). The main pathophysiological feature of proliferative retinopathy, as one of the defining features of diabetes, is VEGF stimulated angiogenesis; hence, experimental therapies target inhibition of VEGF (12). On the other hand, successful therapeutic administration of VEGF was reported in limb ischaemia-another defining feature of diabetes (11). The data of a recent study (2) suggested that VEGF could protect the functional integrity of blood vessels. This event supported the hypothesis that treatment with VEGF at early stages of STZ diabetes could preserve vascular function accompanied by changes in the oxidative environment (2,4). The negative correlation between VEGF and MDA in the present study supported this hypothesis. Plasma VEGF levels were restored after Se administration in STZ-induced DM. Interestingly, it has been reported that high dose selenium down-regulated VEGF production in diabetic retinopathy and epithelial cancer cells (42). This discrepancy was probably due to the different tissue sources of VEGF, different clinical cases, and different doses of selenium used. 

ET1 is a potent vasoactive and mitogenic peptide produced by vascular endothelium. Our results have revealed a significant increase in plasma ET1 concentrations in diabetic rats. Elevated plasma ET1 have been found in clinical (15,16) and experimental diabetes (17,18). The increased ET1 levels found in the present study could be attributed to abnormal production by affected endothelium in conditions related to hyperglycemia and a disturbed oxidant/antioxidant balance (17,18). The presence of a positive correlation between AOPP- ET1 and FRAP-ET1 supported this observation. Se administration did not affect the plasma ET1 levels in our study. A previous study reported that selenium treatment dimished the elevated ET1 levels in thoracic aorta samples obtained from diabetic rats (18). The reason for this discordance probably was due to the short term experimental selenium treatment that changed only tissue ET1, but was not enough to alter plasma ET1 levels. 

## Conclusion

We observed accentuated oxidative stress and impaired endothelial function in STZ-induced diabetes. Se, though its insulin-mimetic actions, was observed to lower plasma glucose levels. Because lipids are the primary target of oxidative stress, it is not surprising that the antioxidant Se initially reduced MDA in early stages of diabetes. The second target of oxidative stress is proteins. The relatively short duration of Se treatment may not reduce AOPP levels. Se administration normalized decreased VEGF levels, therefore it contributed to improvement of endothelial dysfunction in early diabetes. Because of the short duration of the present experiment, a long-term study with higher doses of Se should be performed in order to receive more significant results. 
